# Abnormal bisubclavian trunk arising from the aortic arch determined by cadaver dissection of a native dog: A case report

**Published:** 2015-09-15

**Authors:** Younes Kamali, Mina Tadjalli

**Affiliations:** Department of Basic Sciences, Division of Anatomy and Embryology, School of Veterinary Medicine, Shiraz University, Shiraz, Iran.

**Keywords:** Bisubclavian trunk, Dog, Vascular ring anomaly

## Abstract

Congenital anomalies of the great thoracic vessels have been reported in 20% of dogs and cats. In some cases, the vascular ring anomalies remain unrecognized throughout the lifetime of an animal. This report describes a case with an unusual vascular ring anomaly (VRA) that was detected during dissection on a cadaver of an approximately two-year-old male native mixed breed dog. No history of the animal’s life was available. But, good physical condition and age of the animal based on dentition indicated the anomaly was perhaps asymptomatic. Two main branches of the aorta were identified with the initial branch being a bicarotid trunk followed by a bisubclavian trunk. The left subclavian and aberrant right subclavian arteries formed a very short trunk and arose directly from the aortic arch. No dilatation cranial to the esophageal sulcus was found. To the authors' knowledge, our case is the first report of such anomalies perhaps without any clinical signs in a native dog in Iran.

## Introduction

From the convexity of the aortic arch arise the left subclavian artery and brachiocephalic trunk. Brachiocephalic trunk extends cranially and to the right from the aortic arch ventral to the trachea. Brachiocephalic trunk divides into two common carotid arteries and the right subclavian artery.^1^ Vascular ring anomalies (VRA) are a relatively rare genetic cardiovascular disorder of dogs characterized by abnormal persistent of the fetal aortic arch.^2^ Persistent right aortic arch accounts for up to 95% of VRA in dogs.^3^ Other VRA include aberrant left and right subclavian arteries, double aortic arch, right patent ductus arteriosus or right-sided ligamentum arteriosum, persistent right dorsal aorta, aberrant intercostal arteries.^4^ Anomalies in the development of the aortic arch arteries can lead to partial or complete vascular rings around the esophagus and trachea at the base of the heart.^5^ Among the breeds of dogs, German shepherds, Greyhounds, Irish setters and Boston terriers are most predisposed to this vascular ring anomaly.^5^

## Case Description

**Fig. 1:**
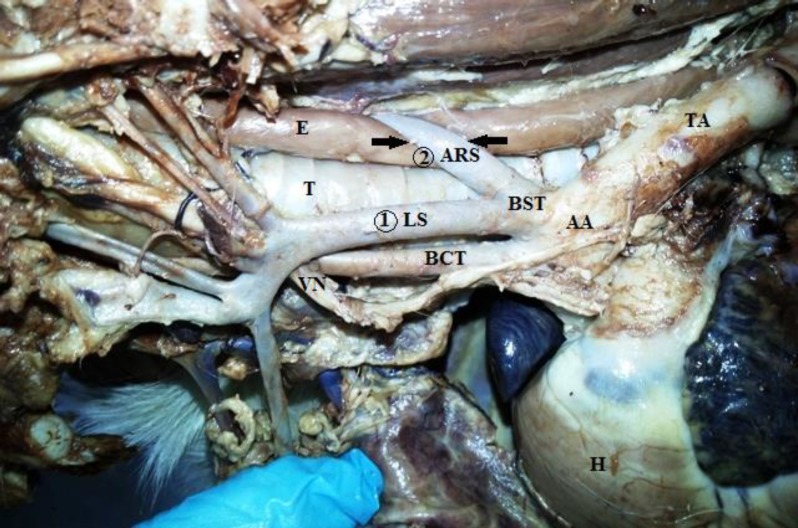
Left lateral view of the mediastinal cavity depicting (H) Heart; (AA) Aortic arch; (BCT) Bicarotid trunk; (BST) Bisubclavian trunk; (1:LS) Left subclavian artery; (2:ARS) Aberrant right subclavian artery; (TA) Thoracic aorta; (VN) Vagus nerve; and (T) Trachea. The arrows show the passage site of the ARS artery dorsal to the esophagus (E).

During dissection on the cadaver of an approximately two-year-old male mixed breed dog for veterinary students, an unusual VRA was accidentally observed. No history of the animal’s life was available. But, good physical condition and age of the animal based on dentition indicated the anomaly was perhaps asymptomatic. The surrounding connective tissues were carefully removed to provide a clear visual field for observing the great vessels of the aortic arch. Two main branches of the aorta were identified with the initial branch being a bicarotid trunk followed by a bisubclavian trunk (Fig. 1). 

**Fig. 2:**
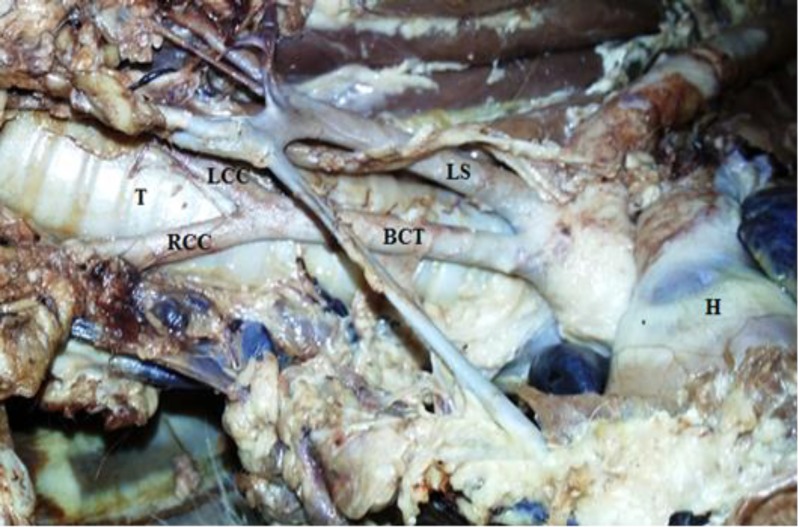
Left ventrolateral aspect of the heart and surrounding vasculature showing bifurcation of the bicarotid trunk (BCT) into the left and right common carotid arteries (LCC and RCC), (T) Trachea; (H) Heart.

The bicarotid trunk was originated from the aortic arch at the ventrolateral aspect of the trachea and after a 4 cm course cranially, at the level of the first rib divided into the left and right common carotid arteries (Fig. 2). 

**Fig. 3:**
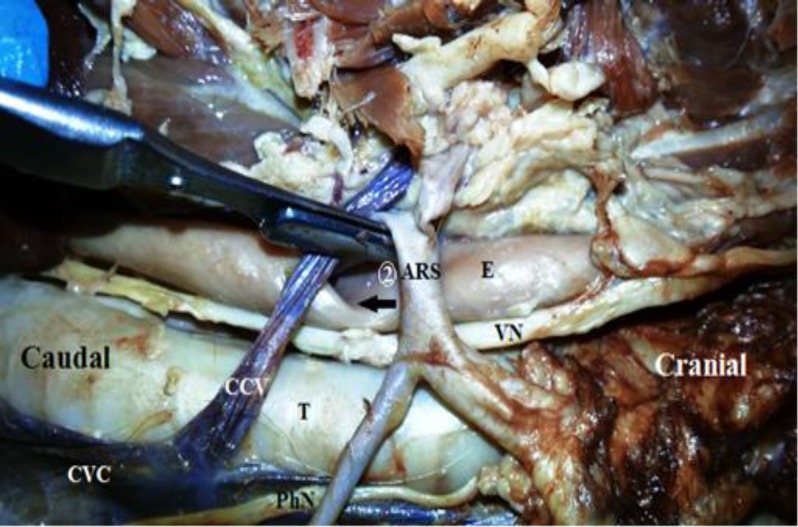
Right lateral view of the mediastinal cavity showing (2:ARS) Aberrant right subclavian artery; (CVC) Cranial vena cava; (CCV) Costocervical vein; (VN) Vagus nerve; (PhN) Phrenic nerve; and (T) Trachea. Note the sulcus (arrow) on the dorsal aspect of the esophagus (E).

Afterwards, the left subclavian and aberrant right subclavian arteries formed a very short trunk and arose directly from the aortic arch at a distance of 2 mm above the origin of the former trunk (Fig. 1). The aberrant right subclavian artery departed the left side of the mediastinum and crossed dorsal to the esophagus to the right side (Fig. 3). Both subclavian arteries branched off the usual branches. The dorsal aspect of the esophagus was constricted by the anomalous artery. However, no dilatation cranial to the sulcus was formed (Fig. 3). 

## Discussion

Vascular rings are developmental malformations of the great thoracic vessels and are the most common cause of esophageal constriction. Similarly, the anomalous origin of the right subclavian artery can result in a vascular ring around the esophagus causing megaesophgus cranial to the stricture.^3^^,^^4^ In such cases, the right subclavian artery arises directly from the aortic arch instead of from the brachiocephalic trunk. In terms of embryonic origin, the anomaly develops when the right dorsal aorta, between the fourth aortic arch and the seventh dorsal inter-segmental artery, disappears while the part caudal to it (which normally regresses) remains. As a consequence, the right subclavian artery consists of the remnant of the right dorsal aorta caudal to the seventh dorsal intersegmental artery and the intersegmental artery itself.^6^ In most reported cases of such anomaly, the aberrant right subclavian artery arises separately from the aortic arch.^3^^,^^7^^-^^9^ But, in our case as already mentioned, the two subclavian arteries formed a short common trunk.

 According to the veterinary literatures, the most predisposed to this condition are purebred dogs.^5^ But, in the present case, the cadaver is an Iranian mixed breed dog. To the authors' best knowledge, this is the first case report describing an abnormal bisubclavian trunk perhaps without any clinical signs in a native dog.
